# TRIM25 promotes Capicua degradation independently of ERK in the absence of ATXN1L

**DOI:** 10.1186/s12915-020-00895-0

**Published:** 2020-10-28

**Authors:** Derek Wong, Lisa Sogerer, Samantha S. Lee, Victor Wong, Amy Lum, Adrian B. Levine, Marco A. Marra, Stephen Yip

**Affiliations:** 1 Pathology and Laboratory Medicine, University of British Columbia, Vancouver, Canada; 2 Molecular Oncology, BC Cancer Agency, Vancouver, Canada; 3 Department of Electrical and Computer Engineering, Technical University of Munich, Munich, Germany; 4 Department of Biological and Chemical Engineering, University of British Columbia, Vancouver, Canada; 5 Molecular Biology and Biochemistry, Simon Fraser University, Burnaby, Canada; 6 Canada’s Michael Smith Genome Sciences Centre, BC Cancer, Vancouver, Canada; 7 Vancouver General Hospital, Vancouver, Canada

**Keywords:** Capicua, CIC, ATXN1L, MAPK, Ubiquitin, Proteasomal degradation, TRIM25, Glioblastoma, Breast carcinoma

## Abstract

**Background:**

Aberrations in *Capicua* (*CIC*) have recently been implicated as a negative prognostic factor in a multitude of cancer types through the derepression of targets downstream of the mitogen-activated protein kinase (MAPK) signaling cascade, such as oncogenic E26 transformation-specific (ETS) transcription factors. The Ataxin-family protein ATXN1L has previously been reported to interact with CIC in both developmental and disease contexts to facilitate the repression of CIC target genes and promote the post-translational stability of CIC. However, little is known about the mechanisms at the base of ATXN1L-mediated CIC post-translational stability.

**Results:**

Functional in vitro studies utilizing *ATXN1L*^KO^ human cell lines revealed that loss of ATXN1L leads to the accumulation of polyubiquitinated CIC protein, promoting its degradation through the proteasome. Although transcriptomic signatures of *ATXN1L*^KO^ cell lines indicated upregulation of the mitogen-activated protein kinase pathway, ERK activity was found to contribute to CIC function but not stability. Degradation of CIC protein following loss of ATXN1L was instead observed to be mediated by the E3 ubiquitin ligase TRIM25 which was further validated using glioma-derived cell lines and the TCGA breast carcinoma and liver hepatocellular carcinoma cohorts.

**Conclusions:**

The post-translational regulation of CIC through ATXN1L and TRIM25 independent of ERK activity suggests that the regulation of CIC stability and function is more intricate than previously appreciated and involves several independent pathways. As CIC status has become a prognostic factor in several cancer types, further knowledge into the mechanisms which govern CIC stability and function may prove useful for future therapeutic approaches.

## Background

*Capicua* (*CIC*), a member of the high-mobility group (HMG) box protein superfamily, is a highly conserved nuclear sensor of receptor tyrosine kinase (RTK) activation. CIC was first identified in *Drosophila melanogaster* as a regulator of growth and proliferation of specific tissues during development and exists as two isoforms (CIC-S and CIC-L) [1–6]. In general, CIC acts as a repressor of receptor tyrosine kinase (RTK)-responsive genes and is inactivated through phosphorylation by ERK, a member of the mitogen-activated protein kinase (MAPK) cascade [2, 5–8]. The most well-characterized mammalian targets of CIC are the oncogenic E26 transformation-specific (ETS) transcription factor family genes *ETV1/4/*5 which promote cell proliferation, motility, and invasion, downstream of RTK.

Mutations in *CIC* were first identified in type I low-grade glioma (LGG) (50–70%), a subtype of LGG defined by simultaneous IDH mutation and 1p19q codeletion [9–11]. More recently, dysregulation of CIC activity has been associated with negative prognostic features and/or oncogenesis in several human cancer types [12] including sarcoma [13], glioblastoma [14], hepatocellular carcinoma [15] lung adenocarcinoma [16], and pancreatic adenocarcinoma [17]. This may be due to the subsequent upregulation of RTK-responsive genes such as the oncogenic ETS transcription factors (*ETV1/4/5*) [15–23]. In addition to the loss of function mutations, post-translational dysregulation of CIC has also been reported in multiple contexts. In glioblastoma, CIC inactivation occurs as a result of ERK phosphorylation and subsequent degradation through interaction with the E3-ligase PJA1 [14] or promotes nuclear export by the kinase c-Src [24]. Conversely, dysregulation of CIC through loss of ATXN1L has been observed in several other cancer subtypes including low-grade glioma and prostate, stomach, pancreatic, gastric, and lung adenocarcinomas [17, 25].

ATXN1L is a member of the Ataxin protein family which has been shown to be a potent regulator of CIC function in both development and cancer [17, 25, 26]. Previous studies investigating murine development have indicated some level of functional redundancy between ATXN1L and its homolog ATXN1. However, these studies have also highlighted that loss of ATXN1L affected CIC function much more profoundly and robustly compared to the loss of ATXN1, which is also consistent in cancer [26, 27]. Although several studies have consistently reported that the loss of ATXN1L results in the post-translational dysregulation of CIC stability and function, the exact mechanism responsible for this phenomenon remains unknown.

In this study, we explore the physical relationship between CIC and ATXN1L and interrogate the mechanism responsible for CIC instability following the loss of ATXN1L. We uncover an ERK-independent mechanism whereby the interaction between CIC and ATXN1L protects CIC from degradation by the E3-ligase TRIM25. Utilizing both in vitro gene expression data from genetically modified cell lines and patient-derived gene expression data from The Cancer Genome Atlas (TCGA), TRIM25 and CIC-ATXN1L were found to antagonistically regulate similar gene sets related to the cell cycle.

## Results

### Loss of ATXN1L promotes the proteasomal degradation of CIC

**Fig. 1:**
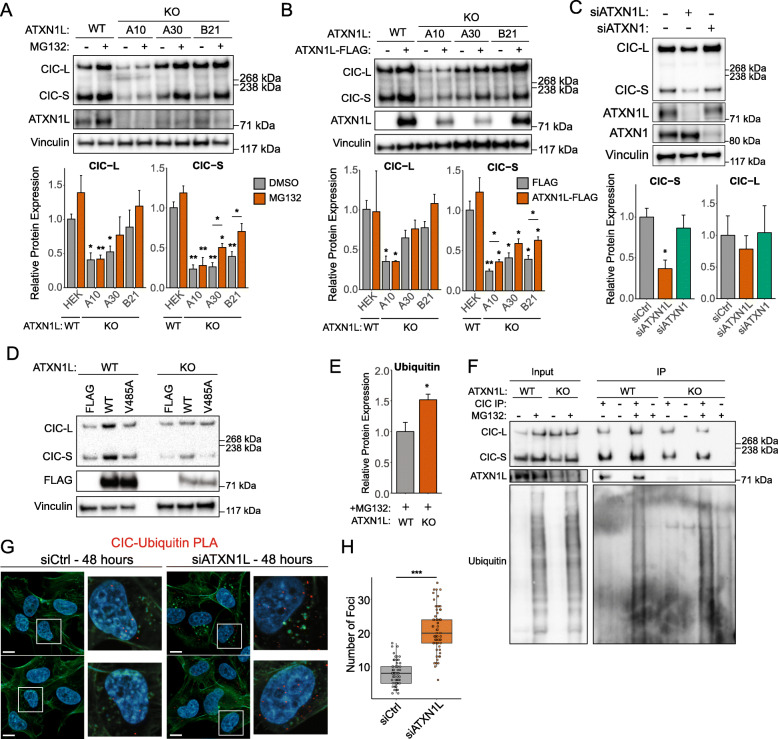
Loss of ATXN1L results in CIC instability. **a** Representative Western blot of *ATXN1L*^WT^ (HEK) and *ATXN1L*^KO^ cell lines (A10, A30, B21) treated with MG132. DMSO was used as a negative control. Below: barplots showing quantification of CIC protein expression. **b** Representative Western blot of *ATXN1L*^WT^ (HEK) and *ATXN1L*^KO^ cell lines (A10, A30, B21), ectopically overexpressing FLAG-tagged ATXN1L. Below: barplots showing quantification of CIC protein expression. **c** Representative Western blot of siRNA knockdown of *ATXN1* and *ATXN1L* in NHA. Scrambled siRNA was used as a negative control. Below: barplots showing quantification of CIC protein expression. **d** Representative Western blot of *ATXN1L*^WT^ (HEK) and *ATXN1L*^KO^ cell lines (A30) ectopically overexpressing FLAG-tagged wild-type ATXN1L or mutant ATXN1L-V485A. **e** Barplots showing quantification of ubiquitin Western blots normalized to total CIC Western blots. **f** Representative Western blot of CIC immunoprecipitation in *ATXN1L*^WT^ (NHA) and *ATXN1L*^KO^ (B82) cell lines treated with MG132. DMSO was used as a negative control. **g** Immunofluorescence images of proximity ligation assay showing CIC-ubiquitin interaction in *ATXN1L*^WT^ (NHA) cell lines following siRNA knockdown of *ATXN1L*. Scrambled siRNA was used as a negative control. White bars denote 10 μm. **h** Tukey boxplots showing quantification of the number of CIC-ubiquitin foci/cell. Western blot quantifications were collected from 3 independent experiments and were normalized to vinculin unless specified otherwise. Error bars represent one standard deviation. PLA quantifications were collected from 65 individual cells. *p* values were calculated using the two-tailed independent Student’s *t* test. Statistically significant values are denoted (*p < 0.05, **p < 0.01, ***p < 0.001). Individual data values can be found in Additional file 17: Table S10

Several studies in human cell lines and mouse studies have reported decreased CIC protein expression following the loss of ATXN1L [17, 25, 26]. In concordance with these studies, decreased CIC protein expression was observed in our *ATXN1L*^KO^ cell lines [25] which could be partially rescued to levels similar to the untreated parental cell line following treatment with MG132, an inhibitor of the 20S subunit of the proteasome (Fig. 1a), or ectopic expression of a FLAG-tagged ATXN1L construct (Fig. 1b). This decrease in CIC protein expression was found to be exclusive to ATXN1L loss and was not observed following siRNA knockdown of *ATXN1*, a homolog of *ATXN1L* (Fig. 1c). Additionally, the introduction of mutant FLAG-tagged *ATXN1L*-V485A, which is unable to interact with CIC [28] (Additional file 1: Figure S1A), also did not rescue CIC protein expression (Fig. 1d). Density gradient fractionation of *ATXN1L*^WT^ and *ATXN1L*^KO^ cell lysates revealed an increase of CIC in higher molecular weight fractions that also contain SUG1, a proteasome subunit (Additional file 1: Figure S1B). Using immunoprecipitation, an increased accumulation of ubiquitin associated with CIC was also observed in *ATXN1L*^KO^ cells (Fig. 1e, f). Similarly, using proximity ligation assay (PLA), the interaction between CIC and ubiquitin was observed to increase following treatment with MG132 in *ATXN1L*^KO^ (Additional file 1: Figure S1C, D) or siRNA knockdown of *ATXN1L* in *ATXN1L*^WT^ cells (Fig. 1g, h).

### ATXN1L-mediated CIC instability is independent of ERK activity

**Fig. 2:**
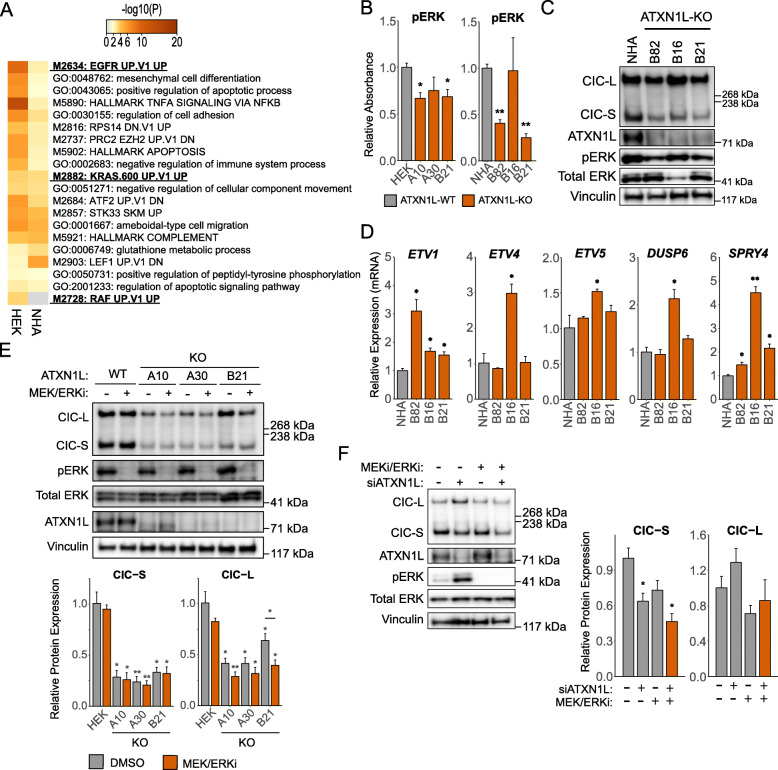
ATXN1L-mediated CIC instability is independent of ERK activity. **a** Heatmap showing the top 20 upregulated gene sets in *ATXN1L*^KO^ NHA and HEK cell lines. Terms related to the MAPK pathway are bolded. **b** ELISA quantification of phosphorylated ERK (pThr202/Tyr204) in *ATXN1L*^WT^ and *ATXN1L*^KO^ NHA and HEK cell lines. Quantifications were normalized to total ERK. **c** Representative Western blot of phosphorylated ERK (pThr202/Tyr204) in *ATXN1L*^WT^ (NHA) and *ATXN1L*^KO^ (B82, B16, B21) cell lines. **d** Relative mRNA expression of CIC target genes *ETV1/4/5*, *DUSP6*, and *SPRY4* in *ATXN1L*^WT^ (NHA) and *ATXN1L*^KO^ (B82, B16, B21) cell lines. Gene expression was normalized to TBP, and the parental *ATXN1L*^WT^ (NHA) cell line was used as a relative control. **e** Representative Western blot of *ATXN1L*^WT^ (HEK) and *ATXN1L*^KO^ (A10, A30, B21) cell lines treated with MEK/ERK inhibitors trametinib/LY3214996. DMSO was used as a negative control. Below: barplot quantifications of CIC expression. Quantifications were normalized to vinculin. **f** Representative Western blot of *ATXN1L*^WT^ (HEK) cells treated with MEK/ERK inhibitor and/or *ATXN1L* siRNA. DMSO and scrambled siRNA were used as negative controls. Right: barplot quantification of CIC expression. Quantifications were normalized to vinculin. Western blot, ELISA, and RT-qPCR quantifications were collected from 3 independent experiments. Error bars represent one standard deviation. *p* values were calculated using the two-tailed independent Student’s *t* test. Statistically significant values are denoted (*p < 0.05, **p < 0.01). Individual data values can be found in Additional file 17: Table S10

Previous studies have established ERK as a downstream component of the mitogen-activated protein kinase (MAPK) pathway important for regulating the protein expression, localization, and function of CIC through phosphorylation [2, 6–8, 14, 29]. Additionally, transcriptomic analyses of *ATXN1L*^KO^ cell lines and The Cancer Genome Atlas (TCGA) patient samples have shown upregulation of gene signatures related to MAPK signaling (Fig. 2a) [25]. To further interrogate the mechanism of CIC degradation following *ATXN1L* loss, we investigated whether ERK activity contributes to the regulation of CIC function in relation to *ATXN1L* status. Phosphorylated ERK (pThr202/Tyr204) was found to be decreased in our *ATXN1L*^KO^ cell lines compared to the *ATXN1L*^WT^ cell lines, detected using ELISA (Fig. 2b) and Western blot (Fig. 2c). Despite the lack of increased ERK activity, *ATXN1L*^KO^ cell lines displayed mRNA upregulation of the many CIC target genes (*ETV1/4/5*, *DUSP6*, *SPRY4*) which are downstream of ERK signaling (Fig. 2d), consistent with previously published data [25]. Further, dual inhibition of MEK and ERK using the small molecule inhibitors trametinib and LY3214996, respectively, did not rescue CIC protein expression in *ATXN1L*^KO^ cell lines (Fig. 2e) or following siRNA knockdown of *ATXN1L* in *ATXN1L*^WT^ cell lines (Fig. 2f).

### Inactivation of CIC by ERK remains intact and independent of ATXN1L

**Fig. 3:**
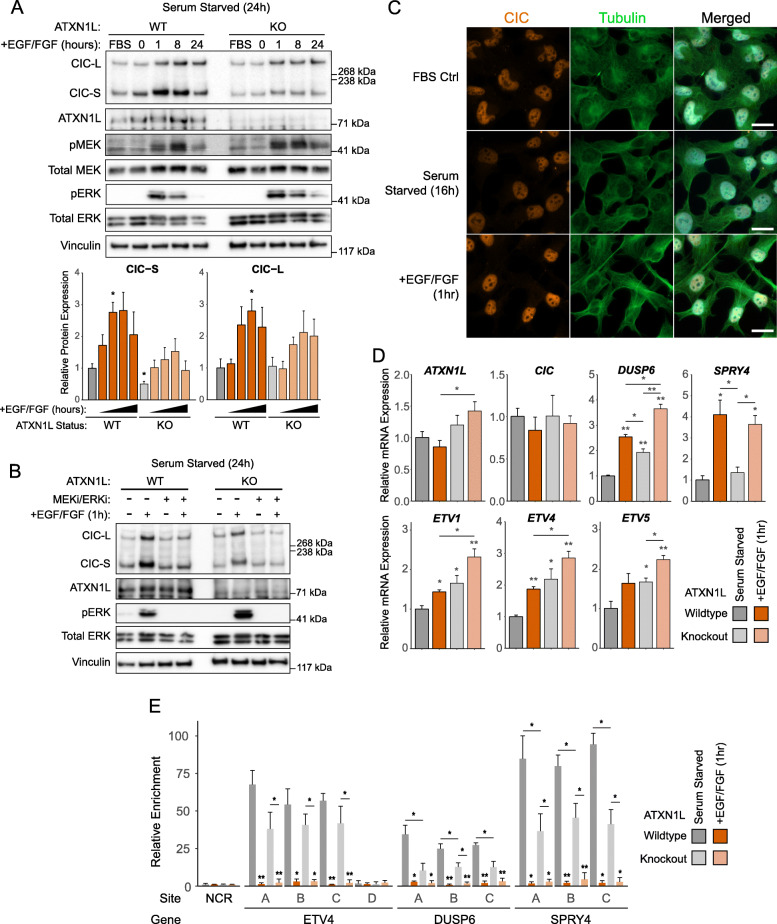
ERK activity relieves CIC’s repressive function. **a** Representative Western blot of *ATXN1L*^WT^ (HEK) and *ATXN1L*^KO^ (A30) cell lines treated with FGF/EGF over 0–24 h following serum starvation. FBS control was cultured in FBS for the duration of the time course. Below: barplot quantifications of CIC expression. Quantifications were normalized to vinculin. **b** Representative Western blot of *ATXN1L*^WT^ (HEK) and *ATXN1L*^KO^ (A30) cell lines treated with FGF/EGF and/or MEK/ERK inhibitors trametinib/LY3214996. **c** Immunofluorescence images of NHA cells cultured in FBS, serum starvation, and EGF/FGF. White bars denote 10 μm. **d** Relative mRNA expression of *ATXN1L*, *CIC*, and CIC target genes *DUSP6*, *SPRY4*, and *ETV1/4/5* in *ATXN1L*^WT^ (NHA) and *ATXN1L*^KO^ (B82) cell lines treated with FGF/EGF for 8 h following serum starvation. Gene expression was normalized to TBP, and the serum-starved parental *ATXN1L*^WT^ (NHA) cell line was used as a relative control. **e** ChIP-PCR showing relative enrichment of the CIC-DNA interaction following serum starvation or treatment with EGF/FGF (1 h) in *ATXN1L*^WT^ (HEK) and *ATXN1L*^KO^ (A30) cell lines. Relative enrichment was normalized to a control region (NCR). Western blot, RT-qPCR, and ChIP-PCR quantifications were collected from 3 independent experiments. Error bars represent one standard deviation. *p* values were calculated using the two-tailed independent Student’s *t* test. Statistically significant values are denoted (*p < 0.05, **p < 0.01). Individual data values can be found in Additional file 17: Table S10

Despite observing no involvement of ERK in the reduction of CIC protein expression in *ATXN1L*^KO^ cells, we were interested to determine if ERK-mediated CIC dysregulation remained intact in our cell system. Treatment of *ATXN1L*^WT^ and *ATXN1L*^KO^ cells with epidermal growth factor (EGF) and fibroblast growth factor (FGF) following serum starvation resulted in an increase of CIC protein expression in both *ATXN1L*^WT^ and *ATXN1L*^KO^ cells (Fig. 3a, Additional file 2: Figure S2A, B), which could be rescued with dual MEK/ERK inhibition (Fig. 3b, Additional file 2: Figure S2C). CIC localization was also not affected by EGF/FGF treatment as observed using immunofluorescence (Fig. 3c). Although CIC protein expression was observed to increase in our cell systems, derepression of several CIC target genes (*ETV1/4/5*, *DUSP6*, *SPRY4*; Fig. 3d, Additional file 2: Figure S2D, E) and decreased CIC binding to the promoter region of target genes (*ETV4*, *DUSP6*, *SPRY4*; Fig. 3e) were observed following EGF/FGF treatment. The increase in CIC protein expression in our cell systems following EGF/FGF treatment was unexpected and thus were further validated in glioblastoma (GBM) cell lines (Additional file 3: Figure S3A, B) and primary brain tumor-initiating cells (BTIC; Additional file 3: Figure S3C).

### CIC degradation is mediated by the E3-ligase TRIM25

**Fig. 4:**
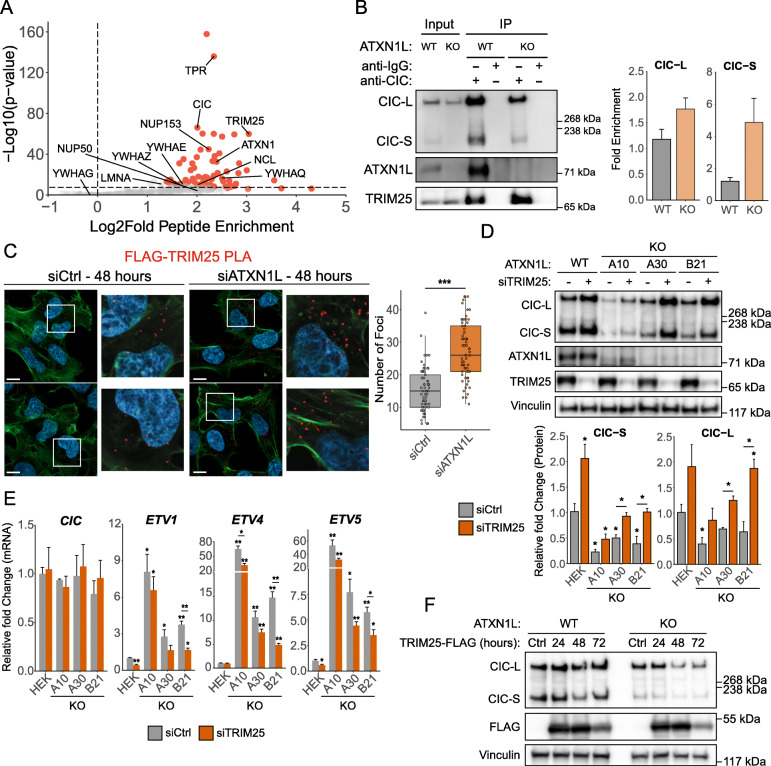
CIC interacts with the E3-ligase TRIM25. **a** Volcano plot showing CIC-interacting proteins identified using CIC immunoprecipitation followed by mass spectrometry in *ATXN1L*^KO^ NHA cells. Red data points are high confidence interactors. **b** Representative Western blot of CIC immunoprecipitation showing interaction with TRIM25 in *ATXN1L*^WT^ (NHA) and *ATXN1L*^KO^ (B82) cell lines. Right: barplot showing quantifications of TRIM25 Western blots. Quantifications were normalized to CIC Western blots. **c** Immunofluorescence images of proximity ligation assay showing FLAG-tagged CIC-S-TRIM25 interaction in *ATXN1L*^WT^ cells treated with *ATXN1L* siRNA. Scrambled siRNA was used as a negative control. White bars denote 10 μm. Right: Tukey boxplots showing quantification of the number of FLAG-TRIM25 foci/cell. **d** Representative Western blot of *ATXN1L*^WT^ (HEK) and *ATXN1L*^KO^ (A10, A30, B21) cell lines treated with *TRIM25* siRNA. Scrambled siRNA was used as a negative control. Below: barplot quantifications of CIC protein expression. Quantifications were normalized to vinculin. **e** Relative mRNA expression of *CIC* and CIC target genes *ETV1/4/5* following treatment with *TRIM25* siRNA for 48 h. Scrambled siRNA was used as a negative control. Gene expression was normalized to TBP. **f** Representative Western blot of *ATXN1L*^WT^ (NHA) and *ATXN1L*^KO^ (B82) cell lines ectopically overexpressing FLAG-tagged TRIM25. Empty FLAG vector was used as a negative control. Western blot and RT-qPCR quantifications were collected from 3 independent experiments. PLA quantifications were collected from 65 individual cells. Error bars represent one standard deviation. *p* values were calculated using the two-tailed independent Student’s *t* test. Statistically significant values are denoted (*p < 0.05, **p < 0.01, ***p < 0.001). Individual data values can be found in Additional file 17: Table S10

To further investigate CIC interactors which may be responsible for mediating the degradation of CIC protein following ATXN1L loss, CIC immunoprecipitation was performed in *ATXN1L*^KO^ cell lines treated with MG132, and interacting proteins were identified using mass spectrometry. Previously identified CIC interactors such as 14-3-3 regulatory proteins and the ATXN1L homolog ATXN1 were highly enriched (Fig. 4a, Additional file 4: Table S1). Using *CIC*^KO^ cell lines with stably expressing FLAG-tagged CIC-S [30], CIC interaction with 14-3-3 regulatory proteins and the nuclear pore protein TPR was observed to increase following siRNA knockdown of *ATXN1L* using PLA (Additional file 5: Figure S4A, B, C). CIC was not found to interact with many previously identified proteins involved in transcriptional regulation such as SIN3A, HDAC1/2, and members of the SWI/SNF complex in our *ATXN1L*^KO^ background which may contribute to the dysregulation of CIC target genes [30–32]. Among the most highly enriched CIC interactors was the E3-ligase TRIM25. CIC interaction with TRIM25 was validated using immunoprecipitation (Fig. 4b, Additional file 5: Figure S4D) and PLA (Fig. 4c) and was found to be elevated in both *ATXN1L*^KO^ and *ATXN1L* knockdown cell systems. Knockdown of *TRIM25* using siRNA was able to induce increased protein expression of both CIC isoforms in *ATXN1L*^WT^ and *ATXN1L*^KO^ cells (Fig. 4d) and decrease mRNA expression of CIC target genes *ETV1/4/5* (Fig. 4e). Ectopic overexpression of FLAG-tagged TRIM25 resulted in a further decrease in CIC protein expression in *ATXN1L*^KO^ cells (Fig. 4f). TRIM25 was observed to localize to both the cytoplasm and nucleus (Additional file 5: Figure S4F).

### Validation of CIC-ATXN1L-TRIM25 interactions in additional cell systems

**Fig. 5:**
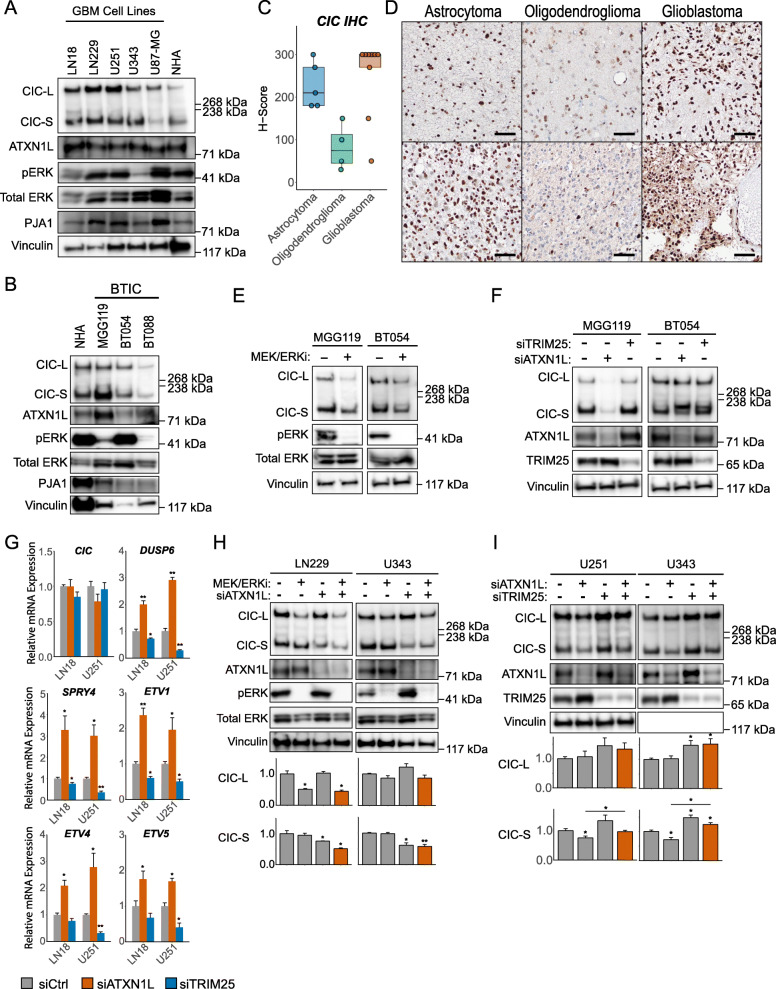
TRIM25 and ATXN1L mediated CIC stability in glioma. **a** Representative Western blot of CIC, ATXN1L, and phosphorylated ERK (pThr202/Tyr204) expression in GBM cell lines. **b** Representative Western blot of CIC, ATXN1L, and phosphorylated ERK (pThr202/Tyr204) expression in BTIC cell lines. **c** Tukey boxplots showing *H*-scores of CIC immunohistochemistry staining on glioma samples. **d** Immunohistochemistry images of CIC staining on glioma samples. Black bars denote 200 μm. **e** Representative Western blot of BTIC cell lines in standard EGF/FGF culture conditions and following 16 h of MEK/ERK inhibition. **f** Representative Western blot of BTIC cell lines following siRNA knockdown of *ATXN1L* or *TRIM25*. Fluorescent RNA was used as a negative control. **g** Relative mRNA expression of *CIC* and CIC target genes (*DUSP6*, *SPRY4*, *ETV1/4/5*) following treatment with *ATXN1L* or *TRIM25* siRNA for 48 h in LN18 and U251 cell lines. Scrambled siRNA was used as a negative control. Gene expression was normalized to TBP. **h** Representative Western blot of LN229 and U343 cell lines treated with MEK/ERK inhibitors trametinib/LY3214996 and/or *ATXN1L* siRNA. DMSO and scrambled siRNA were used as a negative control. Below: barplot quantifications of CIC protein expression. **i** Representative Western blot of U251 and U343 cell lines treated with *ATXN1L* and/or *TRIM25* siRNA. Scrambled siRNA were used as a negative control. Below: barplot quantifications of CIC protein expression. Western blot and RT-qPCR quantifications were collected from three independent experiments. Error bars represent one standard deviation. *p* values were calculated using the two-tailed independent Student’s *t* test. Statistically significant values are denoted (*p < 0.05, **p < 0.01). Individual data values can be found in Additional file 17: Table S10

To further validate our observations, we investigated the relationship between ERK, CIC, ATXN1L, and TRIM25 in additional cellular and biological systems. CIC was found to be expressed in several GBM and primary brain tumor-initiating cell (BTIC) lines (Fig. 5a, b) despite exhibiting increased expression of CIC target genes downstream of MAPK (*ETV1/4/5*, *DUSP6*, *SPRY4*; Additional file 6: Figure S5A, B), compared to NHA cells. Strong expression of CIC was not expected in GBM cell lines as Bunda et al. had previously described ERK-PJA1 mediated degradation of CIC in glioblastoma [14]. Therefore, CIC expression was verified in both low- and high-grade patient glioma samples using immunohistochemistry (*n* = 17) and found to be expressed across all subtypes, with lower expression in oligodendroglioma (Fig. 5c, d). To determine the role of ERK and ATXN1L in regulating CIC protein expression, GBM and BTIC cell lines were treated with dual MEK and ERK inhibition which did not increase CIC expression, consistent with our previous observations (Fig. 5e, Additional file 7: Figure S6A). Similarly, knockdown of *ATXN1L* did result in decreased CIC protein expression in both GBM (Additional file 7: Figure S6B) and BTIC (Fig. 5f) cell lines. Derepression of CIC target genes (*ETV1/4/5*, *DUSP6*, *SPRY4*) was also observed following siRNA knockdown of *ATXN1L* in GBM (Fig. 5g, Additional file 7: Figure S6C) and BTIC (Additional file 7: Figure S6D) cell lines, while siRNA knockdown of *TRIM25* resulted in further repression. Dual inhibition of MEK and ERK was not sufficient to rescue decreases in CIC protein expression following siRNA knockdown of *ATXN1L* (Fig. 5h, Additional file 7: Figure S6E), whereas concurrent knockdown of *ATXN1L* and *TRIM25* was able to rescue decreased CIC protein (Fig. 5i, Additional file 7: Figure S6F).

### *TRIM25* amplification and *CIC* deletion dysregulate similar gene sets

**Fig. 6:**
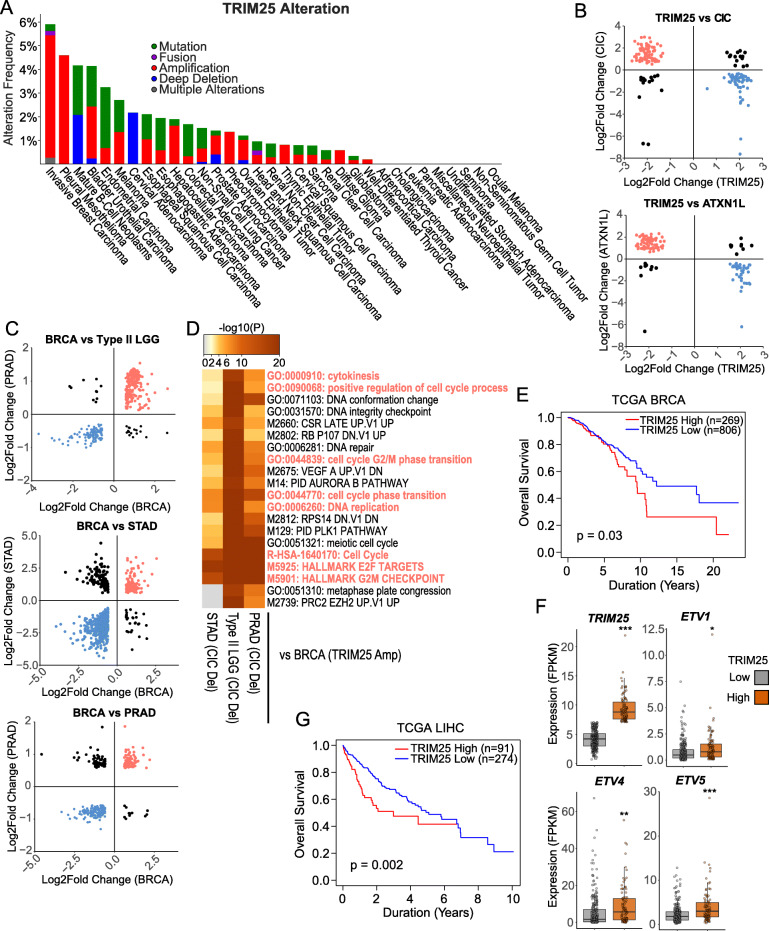
CIC, ATXN1L, and TRIM25 regulate cell cycle in vitro and in TCGA patient data. **a** Barplot displaying the frequency of *TRIM25* alterations in the TCGA Pan-Cancer study. Bars represent a cancer subtype and split based on alteration type. **b** Scatter plot showing the Log2Fold change of differentially expressed genes shared between *TRIM25* siRNA in BT549 and MDA-MB-231 breast cancer cell lines and *CIC/ATXN1L* knockout in NHA cell lines. Differentially expressed genes with directionally discordant change are colored (red/blue). **c** Scatter plot showing the Log2Fold change of differentially expressed genes shared between TCGA BRCA samples with *TRIM25* amplification and TCGA type II LGG, PRAD, and STAD samples with *CIC* deletions. Differentially expressed genes with directionally concordant change are colored (red/blue). **d** Heatmap showing the top 20 enriched gene sets for directionally concordant upregulated differentially expressed genes shared between TCGA BRCA samples with *TRIM25* amplification and TCGA type II LGG, PRAD, and STAD samples with *CIC* deletions. Highlighted terms are terms related to the cell cycle. **e** Kaplan-Meier curve showing the overall survival of TCGA BRCA patients with high (top 25%) and low (bottom 75%) *TRIM25* expression. **f** Tukey barplots showing the expression of *TRIM25*, *ETV1*, *ETV4*, and *ETV5* in TCGA LIHC samples with high (top 25%) and low (bottom 75%) *TRIM25* expression. **g** Kaplan-Meier curve showing the overall survival of TCGA LIHC patients with high (top 25%) and low (bottom 75%) *TRIM25* expression. Statistically significant values are denoted (*p < 0.05, **p < 0.01, ***p < 0.001)

Probing the TCGA database, alterations in *TRIM25* were observed in several cancer types, most notably amplifications in the invasive breast carcinoma (BRCA) and mesothelioma datasets (Fig. 6a). To determine if TRIM25 may regulate the transcription factor functions of CIC, differentially expressed (DE) genes using published gene expression data of *TRIM25* knockdown in breast cancer cell lines (BT549 and MDA-MB-231, Additional file 8: Table S2) from Walsh et al. [33] were compared to DE genes identified in *CIC*^KO^ and *ATXN1L*^KO^ NHA cell lines (Additional file 9: Table S3A, B). As *TRIM25* was knocked down, we expected directional discordance between DE genes identified in *TRIM25* knockdown versus *CIC*^KO^*/ATXN1L*^KO^. Of the 1531 DE genes identified in the *TRIM25* knockdown cell lines (FDR < 0.05, concordant direction), 161 (131 discordant) were shared with *CIC*^KO^ and 122 (104 discordant) were shared with *ATXN1L*^KO^ DE genes (Fig. 6b, Additional file 10: Tables S4A, B). Gene set enrichment analysis of shared discordant genes identified several terms related to cell growth/proliferation and cell attachment/organization which is consistent with CIC/ATXN1L’s function as regulators of the cell cycle and metastasis (Additional file 11: Figure S7, Additional file 12: Table S5A, B). To further validate the relationship between TRIM25 and CIC, differential expression analysis was performed using the TCGA BRCA cohort comparing samples with *TRIM25* amplification versus samples with copy number neutral *TRIM25*. A total of 2789 DE genes were identified (FDR < 0.01, fold change > 1.5; Additional file 13: Table S6A) which were then compared to previously identified DE genes in TCGA type II low-grade glioma (type II LGG, *n* = 1538), prostate adenocarcinoma (PRAD, *n* = 842), and stomach adenocarcinoma (STAD, *n* = 3223) with *CIC* copy number loss [25], expecting directional concordance. Three hundred forty-seven (321 concordant), 290 (221 concordant), and 682 (553 concordant) DE genes were shared between BRCA and type II LGG, PRAD, and STAD, respectively (Fig. 6c, Additional file 13: Table S6B, C, D). Gene set enrichment analysis of upregulated and directionally concordant DE genes shared between BRCA and type II LGG/PRAD/STAD identified several terms related to cell cycle, consistent with previously published work and our cell line analyses (Fig. 6d, Additional file 14: Table S7). BRCA patients which expressed high *TRIM25* expression (top 25%) also showed decreased overall survival (Fig. 6e). In addition to the TCGA BRCA cohort, *TRIM25* was further investigated in the TCGA liver hepatocellular carcinoma (LIHC) cohort in which CIC dysregulation, at the proteomic level, has been described as a negative prognostic factor [15, 34]. Similar to the BRCA cohort, LIHC patients with high *TRIM25* expression (top 25%) were found to have higher expression of the CIC target genes *ETV1/4/5* (Fig. 6f) as well as decreased overall survival (Fig. 6g).

## Discussion

**Fig. 7:**
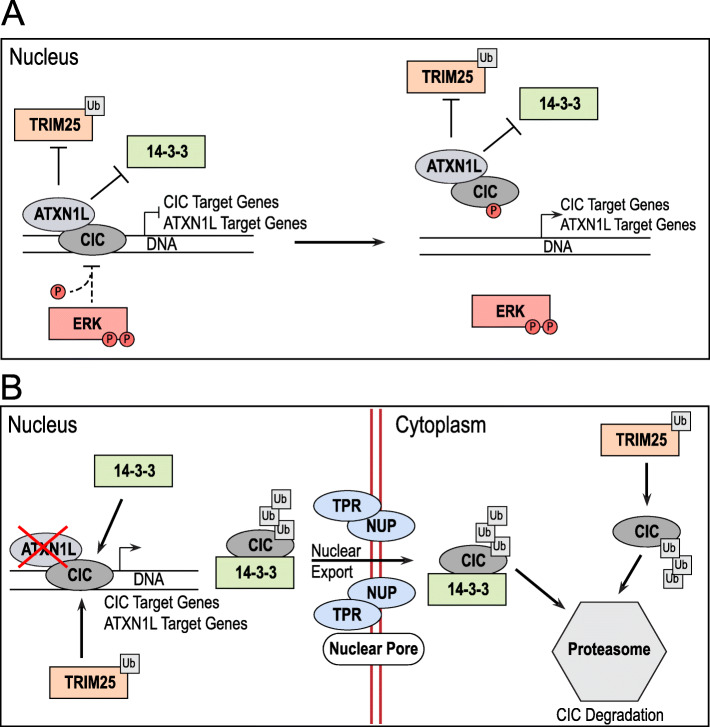
The proposed mechanistic model of the CIC-ATXN1L-TRIM25 interaction. **a** CIC-ATXN1L form a repressive complex which stabilizes CIC from degradation by TRIM25. **b** In the absence of ATXN1L, CIC is targeted by TRIM25 for degradation by ubiquitination and transported for degradation

In this study, we explore the post-translational regulation of CIC in the context of ATXN1L loss and uncover a novel mechanism of CIC protein regulation independent of ERK activity in several cellular systems and contexts (Fig. 7). The role of ATXN1L as a mediator of CIC repressor function has been well established through several functional studies, both in vitro and in vivo [17, 25, 26]; though little has been explored in regard to the mechanisms of ATXN1L-mediated post-translational stability of CIC. Using isogenic *ATXN1L*^KO^ cell lines, CIC instability was observed to be a downstream consequence of increased ubiquitin accumulation and that inhibition of ERK did not affect this CIC instability. Studies investigating the regulation of CIC protein expression in *Drosophila melanogaster* have established ERK as an important mediator of CIC stability through phosphorylation [2, 5–8]. However, unlike mammalian cells, *Drosophila melanogaster* lack an ortholog of *ATXN1L* suggesting that mammalian cells may have evolved several independent pathways to intricately regulate the post-translational function and stability of CIC. This can be evidenced by the temporal differences in CIC inactivation following the loss of ATXN1L (hours) [25] versus phosphorylation by ERK (minutes) [16], which may suggest that CIC instability resulting from the loss of ATXN1L may be due to an increased rate of normal CIC turnover.

In this study, ERK activity was observed to alleviate CIC’s repressive function, but, contrary to several studies, CIC protein expression increased or remained constant. This discrepancy may be due to context-specific and complex regulatory networks involving CIC in mammalian cells that have not been fully explored. As most mammalian studies investigating the role of ERK in regulating CIC stability have been performed in the context of cancers driven heavily by MAPK activation (melanoma, lung adenocarcinoma, glioblastoma), our observations may be due to post-translational feedback loops that are protective against cytotoxic ERK hyperactivation [35] in cell systems that are not heavily driven by or adapted to high levels of MAPK activation. This study also establishes that the regulation of CIC function and protein stability can be regulated by independent pathways which are dependent on the cellular context.

In addition, this study identified the E3-ligase TRIM25, an important mediator of anti-viral activity [36, 37], as a mediator of CIC post-translational stability. Overexpression of *TRIM25* was able to reduce CIC protein expression while siRNA knockdown of *TRIM25* was able to stabilize CIC and promote its tumor suppressor function. Utilizing previously published gene expression data and TCGA cohorts, *TRIM25* amplification in breast carcinoma was found to dysregulate several shared gene sets, related to cell growth and proliferation, with CIC loss. Interestingly, CIC loss has been linked to increased cancer stem cell-like properties in breast cancer cells [38]. Decreased overall survival was also observed in patients with higher *TRIM25* expression in both the BRCA and LIHC TCGA cohorts further supporting the status of CIC function as a prognostic indicator. Although the majority of TRIM25 studies have been focussed on its role as a negative regulator of viral replication, several studies have also suggested that overexpression of TRIM25 may promote tumor progression in hepatocellular carcinoma [39] and lung cancer [40], cell growth and proliferation in prostate adenocarcinoma [41] and breast cancer [42], and metastasis in breast cancer [33], supporting the role of TRIM25 as an oncogene. CIC, which has now been established as a potent tumor suppressor gene, has also been identified as a positive regulator of viral replication [43] which may suggest that the CIC-ATXN1L-TRIM25 axis may be relevant in several different cellular and pathological contexts.

## Conclusions

Here, we describe TRIM25 as an important mediator of CIC post-translational stability, independent of canonical ERK-mediated stability, in both non-transformed, transformed, and primary mammalian cells. Overexpression of *TRIM25* was found to dysregulate similar genes and pathways as *CIC* loss in both in vitro cell lines and TCGA patient samples suggesting that the CIC-ATXN1L-TRIM25 axis may be an important regulator of cell growth and proliferation in several cancer types including breast carcinoma and liver hepatocellular carcinoma.

## Methods

### Immunoprecipitation

For each cell line and replicate, 1–2 15-cm plates at roughly 70–80% confluency were harvested and lysed using Pierce™ IP Lysis Buffer (Thermo Fisher Scientific) supplemented with Halt™ Protease and Phosphatase Inhibitor Cocktail (Thermo Fisher Scientific). Lysates were incubated on ice for 30 min and homogenized by passing through a 25-gauge needle 15–20 times followed by incubation on ice for 30 min. Insoluble cell debris was pelleted down by centrifugation at 13,000 rpm for 15 min at 4 °C. Protein A Dynabeads (Life Technologies) were pre-blocked using 1% BSA, rotating for 1 h at 4 °C. One to 2 mg of protein for each replicate was blocked using 30 μL Protein A Dynabeads (Life Technologies). Conjugation of target antibody to Protein A Dynabeads was performed by adding 1–2.5 μg of antibody to the pre-blocked beads in 1 mL of 1% BSA. Blocked lysate was added to the conjugated beads and incubated, rotating for 3 h at 4 °C. The beads were washed 3× in IP lysis buffer followed by 2× in PBS then suspended in 2× loading buffer and 1× reducing agent. The beads were boiled at 95 °C for 8 min to release captured protein. Antibody information can be found in Additional file 15: Table S8A.

### Cell lysate preparation

Harvested cells were lysed using RIPA buffer (Santa Cruz Biotechnology, CA, USA) supplemented with 1× Halt™ Protease and Phosphatase Inhibitor Cocktail (Thermo Fisher Scientific). Lysates were incubated on ice for 30 min followed by centrifugation at 13,000*g* for 15 min. Protein concentration was quantified using the BCA protein assay (Thermo Fisher Scientific).

### Western blot sample preparation

Samples were normalized using PBS if required followed by the addition of 1× loading buffer (Thermo Fisher Scientific) and 1× reducing agent (Thermo Fisher Scientific) then boiled for 10 min at 95 °C.

### Gel electrophoresis and Western blot

Samples prepared as described above were separated using gel electrophoresis on NuPage 3–8% Tris-Acetate gels run (Life Technologies) in 1× Tris-Acetate running buffer (Life Technologies) or NuPage 10% Bis-Tris gels (Life Technologies) run in 1× MOPS running buffer (Life Technologies). Running buffers were supplemented with oxidizing agent (Life Technologies). Electrophoresis was run at 100 V for 25 min followed by 150 V for 1–2 h. Separated proteins were transferred onto a methanol-activated PVDF membrane (Millipore, Burlington, MA, USA) for 120 min at 100 V in 1× transfer buffer (Life Technologies) with 20% methanol. The membranes were blocked with 5% skim milk in TBS with 0.01% Tween (TBS-T) for 1 h at room temperature followed by overnight incubation with primary antibody. The membranes were incubated with HRP-conjugated secondary antibody (Santa Cruz Biotechnologies) for 1 h at room temperature and washed 3× for 30 min in TBS-T before and after secondary antibody incubation. Protein signal detection was performed using either ECL substrate (Bio-Rad) or SuperSignal West Femto substrate (Thermo Fisher Scientific). Antibody information can be found in Table S1A. Images were captured using an ImageQuant LAS4000 (GE Healthcare Biosciences) or ChemiDoc Imaging System (Bio-Rad), and protein quantifications were performed using the ImageJ software. Antibody information can be found in Additional file 15: Table S8A.

### Cell culture and *ATXN1L* knockout generation

The HEK293a cell line was obtained from Dr. Gregg Morin (Canada’s Michael Smith Genome Sciences Centre, Vancouver, BC, Canada) and authenticated by Genetica DNA Laboratories (Cincinnati, OH, USA). Wild-type IDH1-stable NHA cell line [44] was purchased from Applied Biological Materials (ABM) Inc. (T3022, Richmond, BC, Canada). U251 and U343 glioblastoma cells were obtained from Dr. Shoukat Dedhar (BC Cancer Research Center, Vancouver, BC, Canada), and LN18, LN229, and U87-MG glioblastoma cells were obtained from Dr. Kevin Bennewith (BC Cancer Research Center, Vancouver, BC, Canada). HEK293a, NHA, HOG, U87-MG, U251, and U343 cell lines were cultured in Dulbecco’s modified Eagle’s medium (DMEM; Life Technologies) supplemented with 10% fetal bovine serum (FBS) (Life Technologies) and 5% FBS for LN18 and LN229 cell lines. BTIC cell lines MGG119 [45], BT54, and BT88 [46] were cultured in serum-free Neural Basal Media (Life Technologies) supplemented with N2/B27 (Gibco) and EGF/FGF (20 ng/mL, Peprotech). All cultures were maintained in a 37 °C, 5% CO_2_ incubator. Cells were tested and verified to be mycoplasma-free using MycoFluor™ Mycoplasma Detection Kit (Invitrogen). Unless otherwise stated, cells were washed once with PBS and harvested via scraping or centrifugation at 70–90% confluency. *ATXN1*L^KO^ cell lines were generated using the CRISPR/Cas9 genome editing technology as previously described [25].

### Cell culture transfections

For siRNA knockdown, cells were transfected with Silencer Select (Life Technologies) and Stealth RNAi (Invitrogen) targeted to *ATXN1L*, *ATXN1*, or *TRIM25*. Targeted siRNA information can be found in Additional file 15: Table S8B. Each siRNA was tested independently prior to pooled siRNA experiments (data not shown). Control transfections were performed with Stealth RNAi™ siRNA Negative Control, Med GC (12935300, Thermo Fisher Scientific), or BLOCK-iT Fluorescent Oligo (13750062, Invitrogen). FLAG-tagged ATXN1L (#33242) [47] and FLAG-tagged TRIM25 (#12449) [48] constructs were purchased from Addgene. Transfections were performed at ~ 70% confluency using TransIT-X2® Dynamic Delivery System (Mirus Bio LLC) or nucleofection (Mouse Neural Stem Cell Nucleofector Kit: VPG-1004, Lonza, Basel, Switzerland) according to the manufacturer’s protocol. Cells were harvested 24–72 h post-transfection for experiments. Stable transfection of FLAG-tagged CIC-S in *CIC*^KO^ cells was performed as previously described [30].

### Drug treatments

Cell lines were treated overnight for 16 h with 2–10 μM of MG132 prior to harvesting. LY3214996 was used for ERK inhibition at a concentration of 10 nM. Trametinib was used for MEK inhibition at a concentration of 5 nM. Both drugs were dissolved in DMSO. MEK and ERK inhibition were treated overnight for 16 h or the specified time.

### Immunofluorescence and proximity ligation assay

Cells were seeded and cultured on glass coverslips for 24–72 h and fixed directly to the coverslip using 4% PFA for 20 min at room temperature. Fixed cells were washed in PBS and permeabilized using 0.2% Triton X-100 at room temperature for 20 min. Fixed cells were blocked in 2.5% BSA for 1 h followed by overnight incubation at 4 °C in primary antibody diluted in a blocking solution. Primary antibody information can be found in Additional file 15: Table S8A. Primary treated cells were washed 3× for 5 min with PBS and incubated for 1 h at room temperature with Alexa Fluor 488- or Alexa Fluor 546-conjugated secondary antibody (Invitrogen), diluted in a blocking solution. For proximity ligation assay (PLA), fixation and permeabilization were performed as above and PLA was performed using Duolink® PLA Technology (Sigma) as per the manufacturer’s protocol. Stained cells were washed in PBS, incubated in DAPI (Invitrogen), and diluted in PBS for 5 min. Phalloidin (Invitrogen) staining was performed as per the manufacturer’s protocol. Stained cells were mounted onto a slide using ProLong™ Gold Antifade Mountant (Invitrogen). Images were captured on Zeiss LSM800 confocal microscope or Colibri Upright LED-based microscope using the ZEN microscope software. PLA signals were quantified using ImageJ.

### Immunohistochemistry

Slides were deparaffinized using xylene and a decreasing ethanol gradient. Slides were stained using the Ventana BenchMark automated stainer (Roche, Basel, Switzerland). Normal brain and a normal tissue TMA were used as controls. Antibody dilutions can be found in Additional file 15: Table S8A. Stained slides were blindly scored by two independent neuropathologists.

### RT-qPCR

RNA extraction was performed using the RNeasy Mini Kit (74104, Qiagen) according to the manufacturer’s protocol. One microgram of template RNA was converted to cDNA using the SuperScript™ IV First-Strand Synthesis System (Invitrogen) according to the manufacturer’s protocol. RT-qPCR was performed using 4–12.5 ng of template cDNA using PowerUp™ SYBR™ Green Master Mix (Applied Biosystems) according to the manufacturer’s recommended reaction component amounts and cycling conditions. Primer sequence information can be found in Additional file 15: Table S8C. RT-qPCR reactions were run on a QuantStudio 6 Flex Real-Time PCR System (Thermo Fisher Scientific) using the QuantStudio™ Real-Time PCR software to generate C_T_ values. Analysis of relative mRNA expression was performed using the 2-ΔΔCT method with TATA-box-binding protein (TBP, IDT Technologies) expression as an endogenous control.

### mRNA sequencing and gene expression analyses

Sample quality control was performed using the Agilent 2100 Bioanalyzer. Samples were then prepped following the standard protocol for the NEBnext Ultra ii Stranded mRNA (New England Biolabs). Sequencing was performed on the Illumina NextSeq 500 with paired-end 42 bp × 42 bp reads. De-multiplexed read sequences were then aligned to the reference sequence using STAR aligners [49]. Assembly was estimated using Cufflinks (http://cole-trapnelllab.github.io/cufflinks/) [50] through bioinformatics apps available on the Illumina Sequence Hub. Two independent monoclonal knockouts with 3 biological replicates from different passages were sequenced for each knockout condition. NHA *CIC*^KO^ cell lines were generated as per LeBlanc et al. [19], and NHA *ATXN1L*^KO^ cell lines were generated per above from Wong et al. [25]. si*TRIM25* RNA sequencing data was obtained from Walsh et al. (10.1016/j.celrep.2017.07.052) [33], and only genes with FDR < 0.05 and directional concordance in both cell lines (BT549 and MDA-MB-231) were considered differentially expressed and used for downstream analyses. For TCGA analyses, genomic alteration data was generated/downloaded from the cBioPortal for Cancer Genomics (https://www.cbioportal.org/) [51, 52], accessed on 31 October 2019. TCGA Breast Carcinoma (BRCA) [53] and Liver Hepatocellular Carcinoma (LIHC) [54] RNA sequencing data was downloaded from the Broad Genome Data Analysis Center (GDAC) Firehose (http://firebrowse.org/) [55], accessed on 31 October 2019. Differential expression analysis was performed using DESeq2 [56]. Only BRCA samples with no *TRIM25* copy number variations were considered wild-type (*n* = 449), and BRCA samples with a copy number of 2 or greater were considered amplified (*n* = 73). BRCA samples used for differential expression analysis can be found in Additional file 16: Table S9.

### Functional enrichment analysis

The Metascape software (http://metascape.org) was used to perform functional enrichment analyses using the single and multiple gene lists model [57]. Gene Ontology (GO) biological processes, hallmark gene sets, reactome gene sets, and oncogenic signatures were used for enrichment analyses of all DE genes, with a *p* value cutoff of 0.05 and a minimum enrichment of 1.5.

## Supplementary information

**Additional file 1: Figure S1** Loss of ATXN1L results in CIC instability. A) Representative Western blot of FLAG immunoprecipitation of FLAG-tagged wildtype ATXN1L and mutant ATXN1L-V485A in HEK cells. B) Representative Western blot of cellular fractionation across a sucrose gradient of 20–40% showing localization of CIC. SUG1 was used as a proteasome marker. Quantification of CIC protein/fraction are displayed below. Values were normalized to the cumulative total of CIC in all fractions. C) Immunofluorescence images of proximity ligation assay showing CIC-Ubiquitin interaction in ATXN1L^WT^ (NHA) and ATXN1L^KO^ (B82) cell lines treated with MG132. DMSO was used as negative control. White bars denote 10 μm. D) Tukey boxplots showing quantification of number of CIC-Ubiquitin foci/cell. * PLA quantifications were collected from 65 individual cells. *p*-values were calculated using the two-tailed independent Student’s t-test. Statistically significant values are denoted (* = *p* < 0.05, ** = *p* < 0.01, *** = *p* < 0.001). Individual data values can be found in Additional file 17: Table S10.

**Additional file 2: Figure S2** ERK dysregulates CIC function. A) Representative Western blot of ATXN1L^WT^ (NHA) and ATXN1L^KO^ (B82) cell lines treated with FGF/EGF over 0-24 hours following serum starvation. FBS control was cultured in FBS for the duration of the timecourse. B) Representative Western blot of HOG cell line treated with FGF/EGF over 0-24 hours following serum starvation. FBS control was cultured in FBS for the duration of the timecourse. C) Representative Western blot of ATXN1L^WT^ (NHA) and ATXN1L^KO^ (B82) cell lines treated with FGF/EGF and/or MEK/ERK inhibitors trametinib/LY3214996. D) Relative mRNA expression of ATXN1L, CIC, and CIC target genes DUSP6, SPRY4, and ETV1/4/5 in ATXN1L^WT^ (HEK) and ATXN1L^KO^ (A30) cell lines treated with FGF/EGF for 8 hours following serum starvation. Gene expression was normalized to TBP and the serum starved parental ATXN1L^WT^ (HEK) cell line was used as a relative control. E) Relative mRNA expression of ATXN1L, CIC, and CIC target genes DUSP6, SPRY4, and ETV1/4/5 in HOG cell line treated with FGF/EGF for 8 hours following serum starvation. Gene expression was normalized to TBP and the serum starved parental ATXN1L^WT^ (HEK) cell line was used as a relative control. * RT-qPCR quantifications were collected from 3 independent experiments. Error bars represent one standard deviation. *p*-values were calculated using the two-tailed independent Student’s t-test. Statistically significant values are denoted (* = *p* < 0.05, ** = *p* < 0.01). Individual data values can be found in Additional file 17: Table S10.

**Additional file 3: Figure S3** Validation of ERK-CIC interaction. A) Representative Western blot of GBM cells following serum starvation and EGF/FGF treatment (1 hour). B) Representative Western blot of GBM cells following serum starvation and EGF/FGF treatment (16 hours). C) Representative Western blot of BTIC MGG119 following EGF/FGF starvation (16 hours) and EGF/FGF treatment over 120 minutes. *Individual data values can be found in Additional file 17: Table S10.

**Additional file 4: Table S1** IP-MS result. Proteins identified following CIC IP-MS in ATXN1L-KO NHA cells.

**Additional file 5: Figure S4** CIC interactors. A) Representative Western blot of CIC^KO^ cells with stable FLAG tagged CIC-S reintroduced treated with ATXN1L siRNA over 72 hours. Scrambled siRNA was used as a negative control. B) Immunofluorescence images of proximity ligation assay showing FLAG-tagged CIC-S^-14^-3-3 interaction in NHA-S cells treated with ATXN1L siRNA. Scrambled siRNA was used as a negative control. White bars denote 10μm. Right: Tukey boxplots showing quantification of number of FLAG-14-3-3 foci/cell. C) Immunofluorescence images of proximity ligation assay showing FLAG-tagged CIC-S-TPR interaction in NHA-S cells treated with ATXN1L siRNA. Scrambled siRNA was used as a negative control. White bars denote 10μm. Right: Tukey boxplots showing quantification of number of FLAG-TPR foci/cell. D) Representative Western blot of CIC immunoprecipitation showing interaction with TRIM25 in ATXN1L^WT^ (HEK) and ATXN1L^KO^ (A30) cell lines. E) Immunofluorescence images showing cellular TRIM25 localization in ATXN1L^WT^ (NHA) and ATXN1L^KO^ (B82) cell lines. * PLA quantifications were collected from 65 individual cells. Error bars represent one standard deviation. *p*-values were calculated using the two-tailed independent Student’s t-test. Statistically significant values are denoted (* = *p* < 0.05, ** = *p* < 0.01, *** = *p* < 0.001).

**Additional file 6: Figure S5** Characterization of GBM and BTIC lines. A) Relative mRNA expression of CIC target genes ETV1/4/5, DUSP6, and SPRY4 in GBM cell lines. Expression was normalized to TBP and NHA was used as a relative control. B) Relative mRNA expression of CIC target genes ETV1/4/5, DUSP6, and SPRY4 in BTIC lines. Expression was normalized to TBP and NHA was used as a relative control. Individual data values can be found in Additional file 17: Table S10.

**Additional file 7: Figure S6** Validation of CIC-ATXN1L-TRIM25 Interaction. A) Representative Western blot of GBM cell lines treated with MEK/ERK inhibitors trametinib/LY3214996 for 16 hours. DMSO was used as a negative control. B) Representative Western blot of GBM cell lines treated with ATXN1L siRNA for 48 hours. Scrambled siRNA was used as negative control. C) Relative mRNA expression of CIC and CIC target genes ETV1/4/5, DUSP6, and SPRY4 in GBM cell lines LN229, U343, and U87-MG following siRNA knockdown of ATXN1L or TRIM25 for 48 hours. Expression was normalized to TBP and scrambled siRNA was used as a negative control. D) Relative mRNA expression of CIC and CIC target genes ETV1/4/5, DUSP6, and SPRY4 in BTIC cell lines MGG119 and BT054 following siRNA knockdown of ATXN1L or TRIM25 for 48 hours. Expression was normalized to TBP and fluorescent RNA was used as a negative control. E) Representative Western blot of GBM cell lines LN18, U251, and U87-MG treated with MEK/ERK inhibitors trametinib/LY3214996 and/or ATXN1L siRNA. DMSO and scrambled siRNA were used as negative control. Below: barplot quantifications of CIC protein expression. F) Representative Western blot of GBM cell lines LN18, LN229, and U87-MG treated with ATXN1L and/or TRIM25 siRNA. Scrambled siRNA were used as negative control. Below: barplot quantifications of CIC protein expression. * RT-qPCR and Western blot quantifications were collected from 3 independent experiments. Error bars represent one standard deviation. p-values were calculated using the two-tailed independent Student’s t-test. Statistically significant values are denoted (* = p < 0.05, ** = p < 0.01). Individual data values can be found in Additional file 17: Table S10.

**Additional file 8: Table S2** TRIM25 differentially expressed genes. Differentially expressed genes identified in siTRIM25 vs siCtrl breast carcinoma cell lines. Data from Walsh et al. study [33].

**Additional file 9: Table S3** NHA Differential expression analyses. A) Differentially expressed genes: NHA CIC Wildtype vs CIC knockout. B) Differentially expressed genes: NHA ATXN1L wildtype vs ATXN1L knockout.

**Additional file 10: Table S4** TRIM25 knockdown vs CIC-ATXN1L knockout. A) Comparison of differentially expressed genes: TRIM25 knockdown vs ATXN1L knockout. B) Comparison of differentially expressed genes: TRIM25 knockdown vs CIC knockout.

**Additional file 11: Figure S7** Pathways dysregulated by TRIM25-CIC. Heatmap showing the top 20 enriched gene sets for directionally discordant differentially expressed genes shared between TRIM25 siRNA in BT549 and MDA-MB-231 breast cancer cell lines and CIC/ATXN1L knockout in NHA cell lines. Red terms are upregulated terms related to cell cycle, growth, and proliferation. Blue terms are downregulated terms related to cell structure, organization, and adhesion.

**Additional file 12: Table S5** TRIM25-CIC-ATXN1L gene set enrichment analysis. A) Gene set enrichment analysis of upregulated discordant DE genes shared between TRIM25 vs CIC and TRIM25 vs ATXN1L. B) Gene set enrichment analysis of downregulated discordant DE genes shared between TRIM25 vs CIC and TRIM25 vs ATXN1L.

**Additional file 13: Table S6** BRCA TRIM25 differential expression analyses. A) Differentially Expressed genes comparing BRCA samples with TRIM25 amplification and BRCA samples with copy number neutral TRIM25. B) Differentially Expressed genes shared between BRCA TRIM25 amp and Type II LGG CIC Del. C) Differentially Expressed genes shared between BRCA TRIM25 amp and PRAD CIC Del. D) Differentially Expressed genes shared between BRCA TRIM25 amp and STAD CIC Del.

**Additional file 14: Table S7** TCGA Gene set enrichment analyses. Gene set enrichment analysis of differentially expressed genes shared and directionally concordant between BRCA TRIM25 amp and Type II LGG/PRAD/STAD CIC Del.

**Additional file 15: Table S8** Antibodies and Primers. A) Antibody information. B) siRNA sequences. C) Primer sequences.

**Additional file 16: Table S9** TCGA BRCA Sample. TCGA BRCA Samples used for differential expression analysis (Amplfied vs Wildtype).

**Additional file 17: Table S10** Data values. Individual data values for figures where *n* < 6.

## Data Availability

All data generated or analyzed during this study are included in this published article, its supplementary information files, and publicly available repositories. Supporting data values have also been included in Additional file 17: Table S10. TCGA datasets generated and/or analyzed during the current study are available from https://www.cbioportal.org/ and http://firebrowse.org/. TRIM25 knockdown gene expression data used for analyses are available from Walsh et al. (10.1016/j.celrep.2017.07.052) [33]. Raw sequencing files are also available in the Gene Expression Omnibus (GEO) repository, accession number: GSE140471 (https://www.ncbi.nlm.nih.gov/geo/query/acc.cgi?acc=GSE140471) [58].

## Abbreviations

ATXN1L: Ataxin-1-like
BRCA: Breast carcinoma
BTIC: Brain tumor-initiating cell
CIC: Capicua
ChIP: Chromatin immunoprecipitation
DE: Differentially expressed
DEA: Differential expression analysis
EGF: Epidermal growth factor
ETS: E26 transformation-specific
FGF: Fibroblast growth factor
GBM: Glioblastoma
GSEA: Gene set enrichment analysis
HMG: High-mobility group
IF: Immunofluorescence
IP: Immunoprecipitation
LGG: Low-grade glioma
LIHC: Liver hepatocellular carcinoma
MAPK: Mitogen-activated protein kinase
NHA: Normal human astrocyte
ODG: Oligodendroglioma
PRAD: Prostate adenocarcinoma
PLA: Proximity ligation assay
RTK: Receptor tyrosine kinase
STAD: Stomach adenocarcinoma
TCGA: The Cancer Genome Atlas
TMA: Tissue micro-array
