# Supplementary figures and images for: TRIM25 promotes Capicua degradation independently of ERK in the absence of ATXN1L
Source: BMC Biol. 2020 Oct 28;18:154. doi: 10.1186/s12915-020-00895-0 (PMC7594423; doi:10.1186/s12915-020-00895-0)

**A**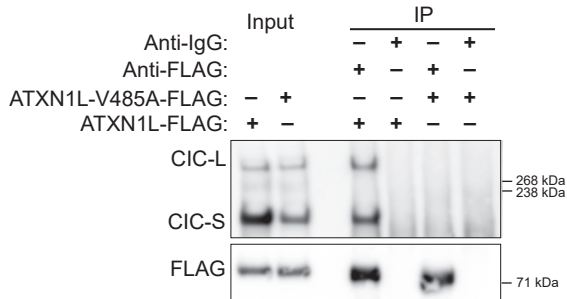**B**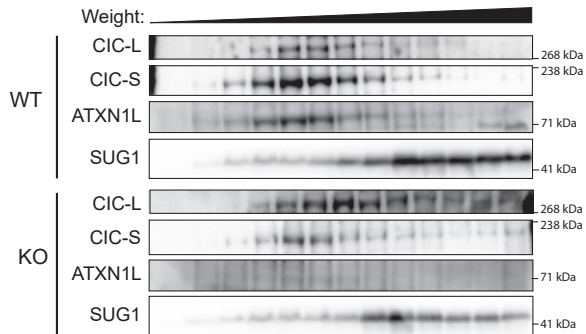**C**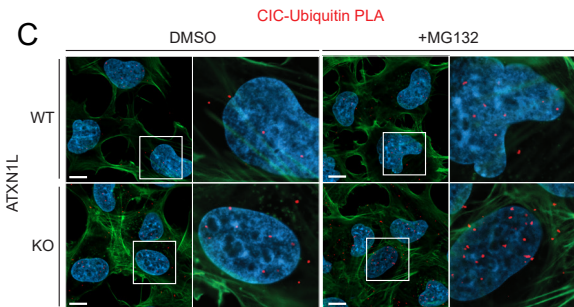**D**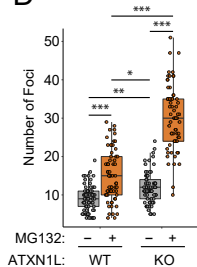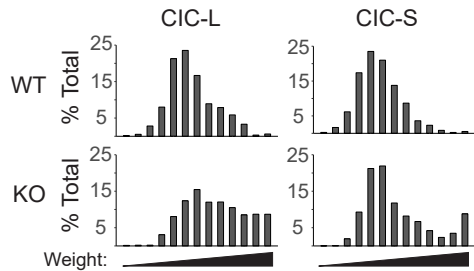

Supplement: Supplementary file 1 — Additional file 1: Figure S1 Loss of ATXN1L results in CIC instability. A) Representative Western blot of FLAG immunoprecipitation of FLAG-tagged wildtype ATXN1L and mutant ATXN1L-V485A in HEK cells. B) Representative Western blot of cellular fractionation across a sucrose gradient of 20–40% showing localization of CIC. SUG1 was used as a proteasome marker. Quantification of CIC protein/fraction are displayed below. Values were normalized to the cumulative total of CIC in all fractions. C) Immunofluorescence images of proximity ligation assay showing CIC-Ubiquitin interaction in ATXN1LWT (NHA) and ATXN1LKO (B82) cell lines treated with MG132. DMSO was used as negative control. White bars denote 10 μm. D) Tukey boxplots showing quantification of number of CIC-Ubiquitin foci/cell. * PLA quantifications were collected from 65 individual cells. p-values were calculated using the two-tailed independent Student’s t-test. Statistically significant values are denoted (* = p < 0.05, ** = p < 0.01, *** = p < 0.001). Individual data values can be found in Additional file 17: Table S10. [file 12915_2020_895_MOESM1_ESM.pdf]

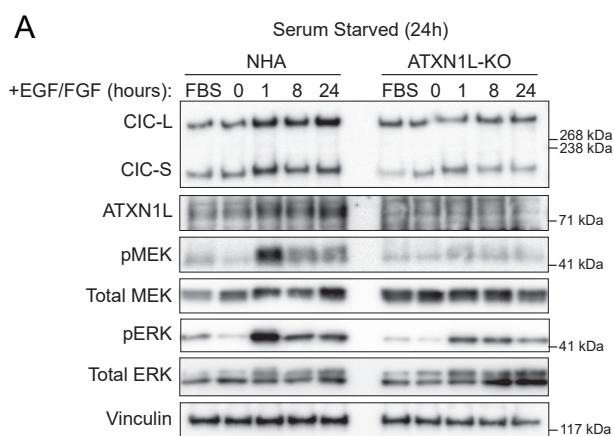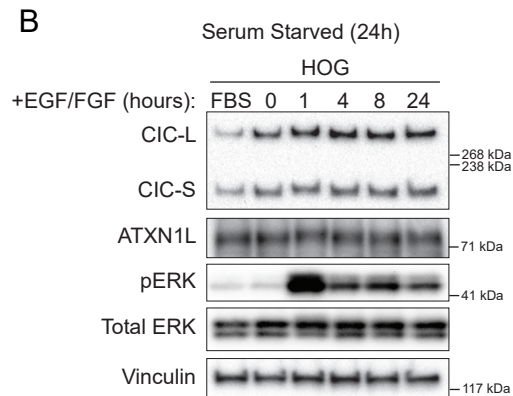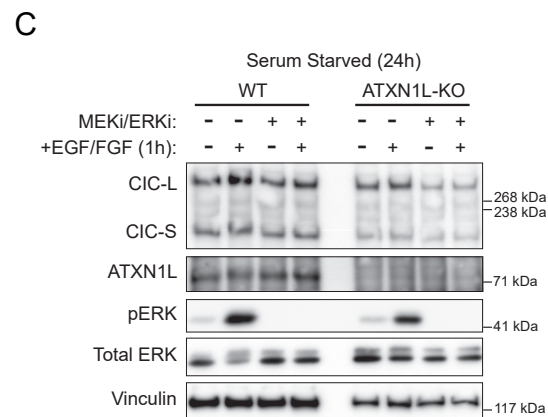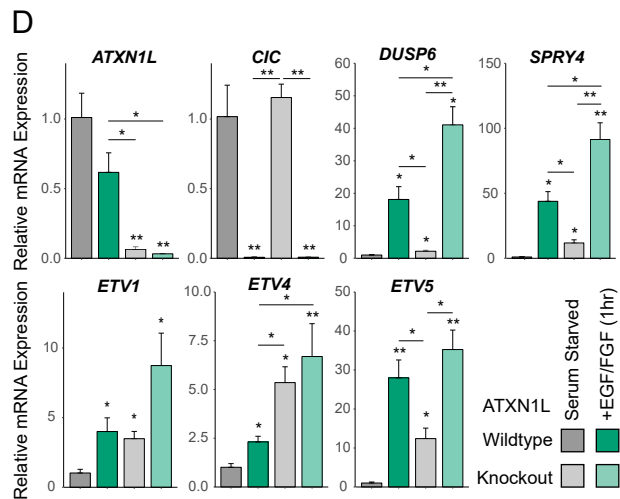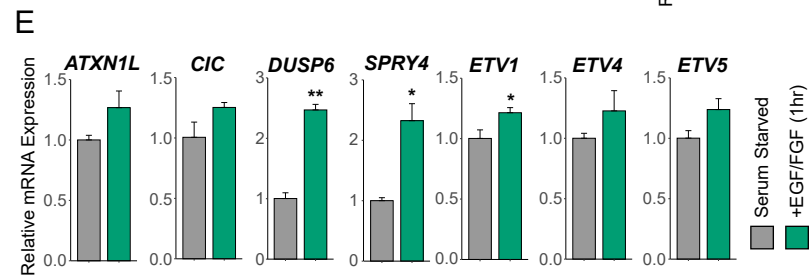

Supplement: Supplementary file 2 — Additional file 2: Figure S2 ERK dysregulates CIC function. A) Representative Western blot of ATXN1LWT (NHA) and ATXN1LKO (B82) cell lines treated with FGF/EGF over 0-24 hours following serum starvation. FBS control was cultured in FBS for the duration of the timecourse. B) Representative Western blot of HOG cell line treated with FGF/EGF over 0-24 hours following serum starvation. FBS control was cultured in FBS for the duration of the timecourse. C) Representative Western blot of ATXN1LWT (NHA) and ATXN1LKO (B82) cell lines treated with FGF/EGF and/or MEK/ERK inhibitors trametinib/LY3214996. D) Relative mRNA expression of ATXN1L, CIC, and CIC target genes DUSP6, SPRY4, and ETV1/4/5 in ATXN1LWT (HEK) and ATXN1LKO (A30) cell lines treated with FGF/EGF for 8 hours following serum starvation. Gene expression was normalized to TBP and the serum starved parental ATXN1LWT (HEK) cell line was used as a relative control. E) Relative mRNA expression of ATXN1L, CIC, and CIC target genes DUSP6, SPRY4, and ETV1/4/5 in HOG cell line treated with FGF/EGF for 8 hours following serum starvation. Gene expression was normalized to TBP and the serum starved parental ATXN1LWT (HEK) cell line was used as a relative control. * RT-qPCR quantifications were collected from 3 independent experiments. Error bars represent one standard deviation. p-values were calculated using the two-tailed independent Student’s t-test. Statistically significant values are denoted (* = p < 0.05, ** = p < 0.01). Individual data values can be found in Additional file 17: Table S10. [file 12915_2020_895_MOESM2_ESM.pdf]

**A**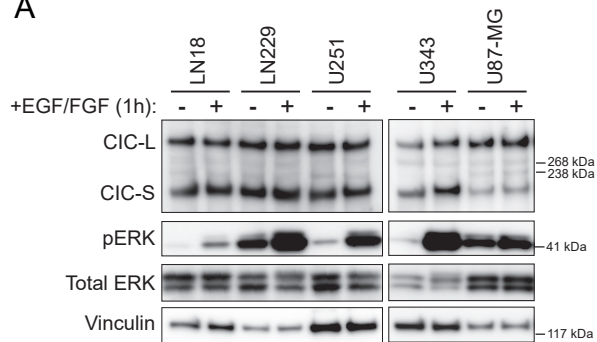**B**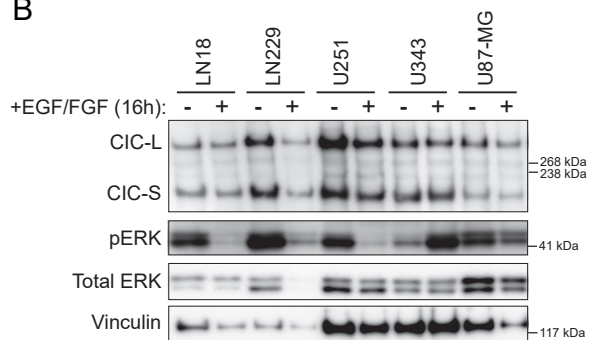**C**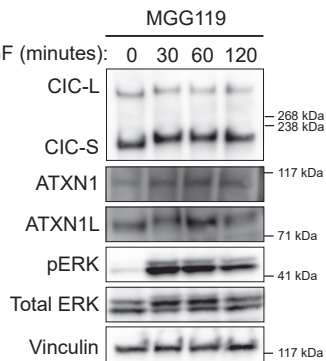

Supplement: Supplementary file 3 — Additional file 3: Figure S3 Validation of ERK-CIC interaction. A) Representative Western blot of GBM cells following serum starvation and EGF/FGF treatment (1 hour). B) Representative Western blot of GBM cells following serum starvation and EGF/FGF treatment (16 hours). C) Representative Western blot of BTIC MGG119 following EGF/FGF starvation (16 hours) and EGF/FGF treatment over 120 minutes. *Individual data values can be found in Additional file 17: Table S10. [file 12915_2020_895_MOESM3_ESM.pdf]

A

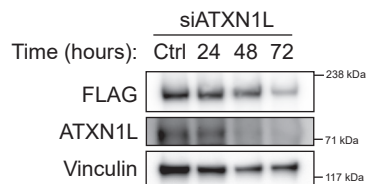

B

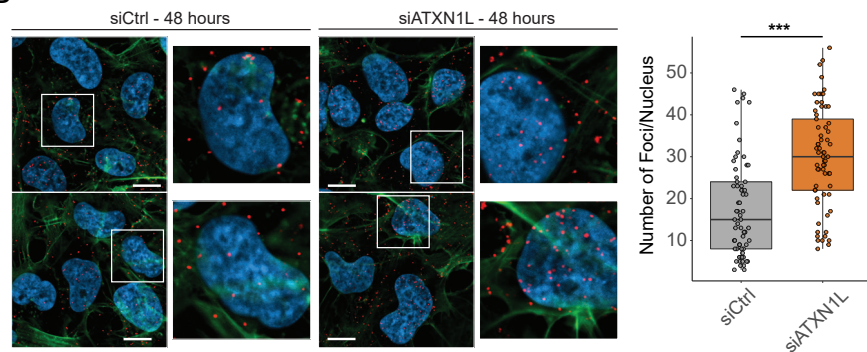

C

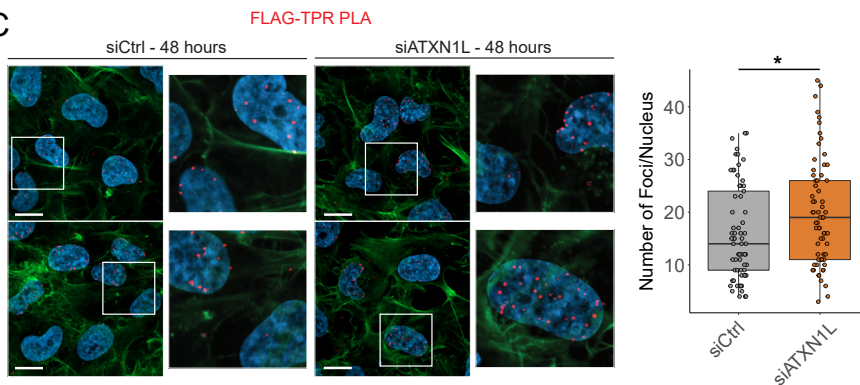

D

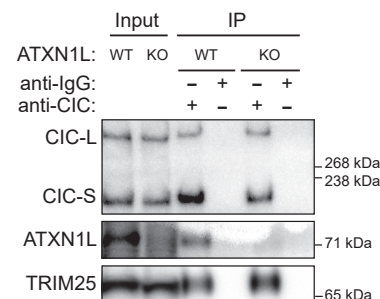

E

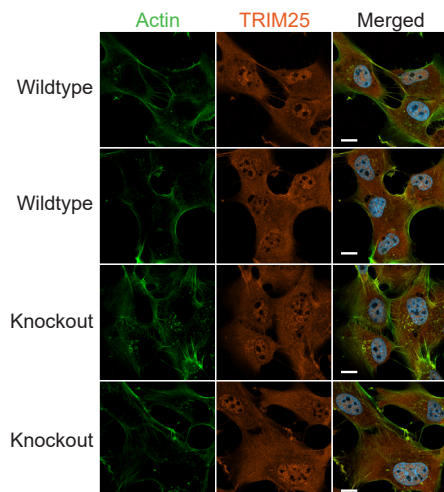

Supplement: Supplementary file 5 — Additional file 5: Figure S4 CIC interactors. A) Representative Western blot of CICKO cells with stable FLAG tagged CIC-S reintroduced treated with ATXN1L siRNA over 72 hours. Scrambled siRNA was used as a negative control. B) Immunofluorescence images of proximity ligation assay showing FLAG-tagged CIC-S-14-3-3 interaction in NHA-S cells treated with ATXN1L siRNA. Scrambled siRNA was used as a negative control. White bars denote 10μm. Right: Tukey boxplots showing quantification of number of FLAG-14-3-3 foci/cell. C) Immunofluorescence images of proximity ligation assay showing FLAG-tagged CIC-S-TPR interaction in NHA-S cells treated with ATXN1L siRNA. Scrambled siRNA was used as a negative control. White bars denote 10μm. Right: Tukey boxplots showing quantification of number of FLAG-TPR foci/cell. D) Representative Western blot of CIC immunoprecipitation showing interaction with TRIM25 in ATXN1LWT (HEK) and ATXN1LKO (A30) cell lines. E) Immunofluorescence images showing cellular TRIM25 localization in ATXN1LWT (NHA) and ATXN1LKO (B82) cell lines. * PLA quantifications were collected from 65 individual cells. Error bars represent one standard deviation. p-values were calculated using the two-tailed independent Student’s t-test. Statistically significant values are denoted (* = p < 0.05, ** = p < 0.01, *** = p < 0.001). [file 12915_2020_895_MOESM5_ESM.pdf]

A

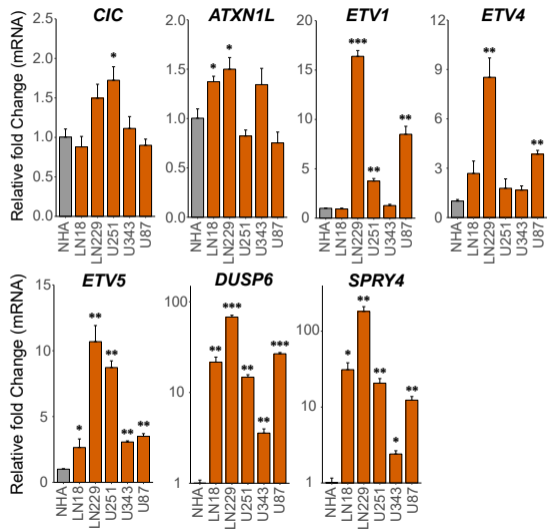

B

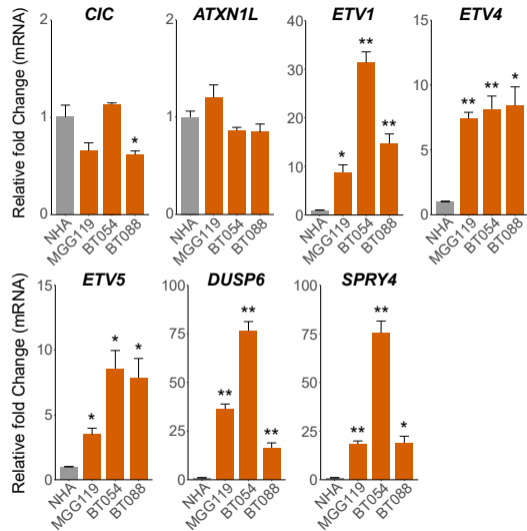

Supplement: Supplementary file 6 — Additional file 6: Figure S5 Characterization of GBM and BTIC lines. A) Relative mRNA expression of CIC target genes ETV1/4/5, DUSP6, and SPRY4 in GBM cell lines. Expression was normalized to TBP and NHA was used as a relative control. B) Relative mRNA expression of CIC target genes ETV1/4/5, DUSP6, and SPRY4 in BTIC lines. Expression was normalized to TBP and NHA was used as a relative control. Individual data values can be found in Additional file 17: Table S10. [file 12915_2020_895_MOESM6_ESM.pdf]

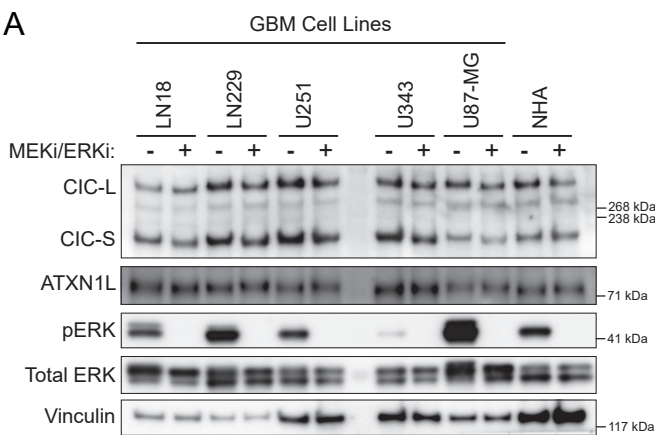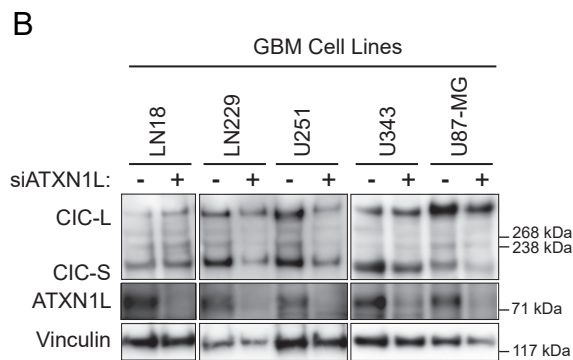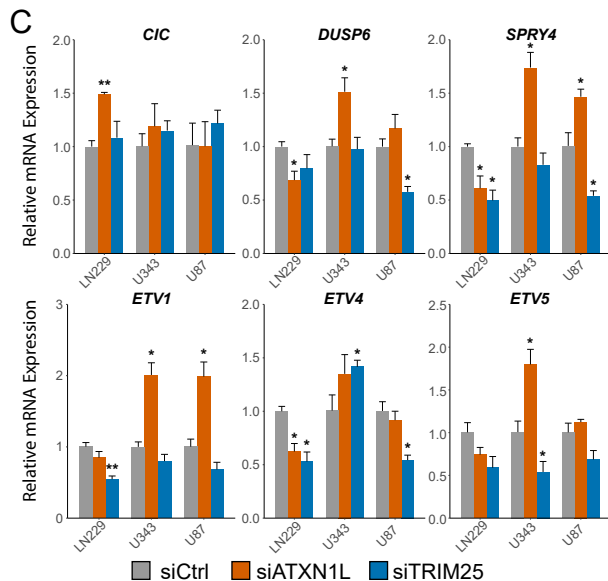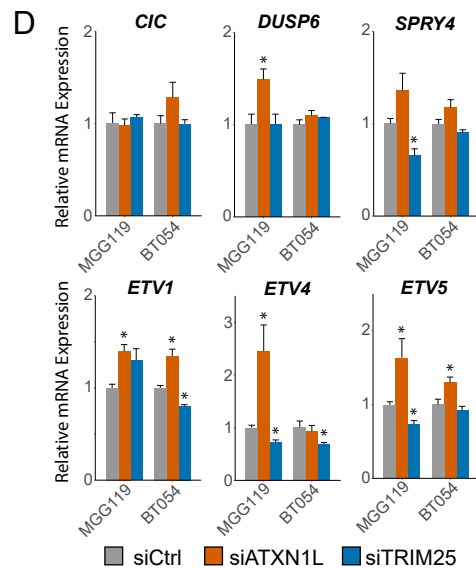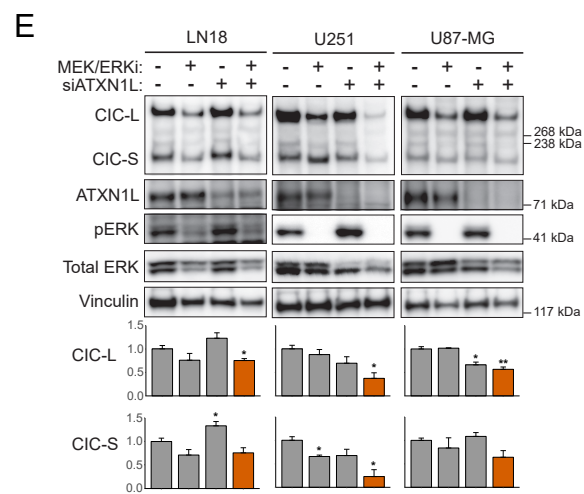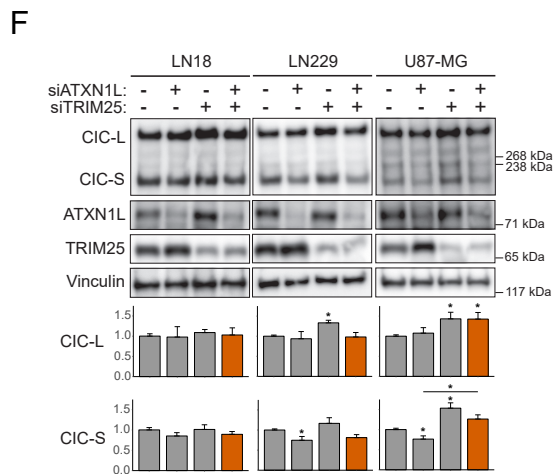

Supplement: Supplementary file 7 — Additional file 7: Figure S6 Validation of CIC-ATXN1L-TRIM25 Interaction. A) Representative Western blot of GBM cell lines treated with MEK/ERK inhibitors trametinib/LY3214996 for 16 hours. DMSO was used as a negative control. B) Representative Western blot of GBM cell lines treated with ATXN1L siRNA for 48 hours. Scrambled siRNA was used as negative control. C) Relative mRNA expression of CIC and CIC target genes ETV1/4/5, DUSP6, and SPRY4 in GBM cell lines LN229, U343, and U87-MG following siRNA knockdown of ATXN1L or TRIM25 for 48 hours. Expression was normalized to TBP and scrambled siRNA was used as a negative control. D) Relative mRNA expression of CIC and CIC target genes ETV1/4/5, DUSP6, and SPRY4 in BTIC cell lines MGG119 and BT054 following siRNA knockdown of ATXN1L or TRIM25 for 48 hours. Expression was normalized to TBP and fluorescent RNA was used as a negative control. E) Representative Western blot of GBM cell lines LN18, U251, and U87-MG treated with MEK/ERK inhibitors trametinib/LY3214996 and/or ATXN1L siRNA. DMSO and scrambled siRNA were used as negative control. Below: barplot quantifications of CIC protein expression. F) Representative Western blot of GBM cell lines LN18, LN229, and U87-MG treated with ATXN1L and/or TRIM25 siRNA. Scrambled siRNA were used as negative control. Below: barplot quantifications of CIC protein expression. * RT-qPCR and Western blot quantifications were collected from 3 independent experiments. Error bars represent one standard deviation. p-values were calculated using the two-tailed independent Student’s t-test. Statistically significant values are denoted (* = p < 0.05, ** = p < 0.01). Individual data values can be found in Additional file 17: Table S10. [file 12915_2020_895_MOESM7_ESM.pdf]

A

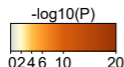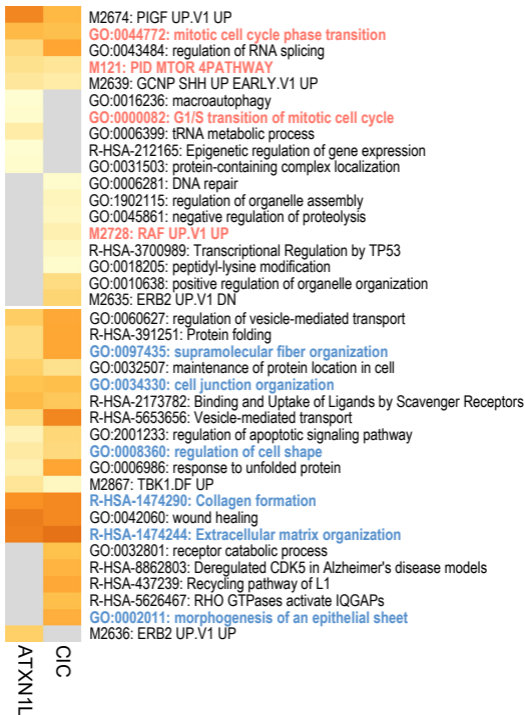

Supplement: Supplementary file 11 — Additional file 11: Figure S7 Pathways dysregulated by TRIM25-CIC. Heatmap showing the top 20 enriched gene sets for directionally discordant differentially expressed genes shared between TRIM25 siRNA in BT549 and MDA-MB-231 breast cancer cell lines and CIC/ATXN1L knockout in NHA cell lines. Red terms are upregulated terms related to cell cycle, growth, and proliferation. Blue terms are downregulated terms related to cell structure, organization, and adhesion. [file 12915_2020_895_MOESM11_ESM.pdf]
